# 492. Impact of the COVID-19 Pandemic on Diabetes Surrogate Markers in a Population of People Living with HIV

**DOI:** 10.1093/ofid/ofab466.691

**Published:** 2021-12-04

**Authors:** Tyler Maxwell, Esther Kanner, Nubriel Hernandez, Suzanne Molino, Jessica E Yager, Shannon Ramdeen, Roopali Sharma

**Affiliations:** 1 SUNY Downstate Medical Center, Brooklyn, New York; 2 Arnold and Marie Schwartz College of Pharmacy, Long Island University, Brooklyn, New York; 3 SUNY Downstate Health Sciences University, Brooklyn, New York; 4 Long Island University, Brooklyn, New York; 5 Brookdale Hospital Medical Center, Brooklyn, New York; 6 Touro College of Pharmacy, New York, NY

## Abstract

**Background:**

COVID-19 has become a worldwide pandemic that brought changes in sociological, economic and health perspectives. The impact of the pandemic on health maintenance is not yet understood, but aspects of the lockdown are being assessed for their impact on society. Diabetes and HIV are diseases that require frequent follow-up to achieve outcomes. Changes to routines during the lockdown, such as physical activity, eating habits, and psychological burden, may result in complications for this patient population.

**Methods:**

This is a multi-center, retrospective cohort study performed between October 2019 to October 2020 at two medical centers in Brooklyn, NY. All adult patients with diagnoses of diabetes and HIV were screened for inclusion. Exclusion criteria included pregnancy and long-term steroid use. Electronic medical records were reviewed to obtain demographic, laboratory data, and appointment retention data. The primary endpoint was the mean change in HbA1c (A1c) values before and after the pandemic. Endpoints were evaluated using paired T-tests and Wilcoxon Sign-Rank tests, where appropriate, and a repeated measures logistic regression model was used to analyze appointment retention rates.

**Results:**

Baseline characteristics are summarized in Table 1. No significance was observed between baseline A1c values and those taken either up to 3 months (p= 0.862) or up to 6 months (p= 0.977) after the start of the pandemic, as shown in Table 2. Similarly, no difference was observed in HIV surrogate markers. A1c significantly decreased from between the 3-month and 6-month study dates, after the start of the pandemic (p= 0.022). Table 3 shows patients were more likely to fulfill a scheduled appointment during the pandemic with an odds ratio of 1.455 (95% CI, 1.119-1.891).

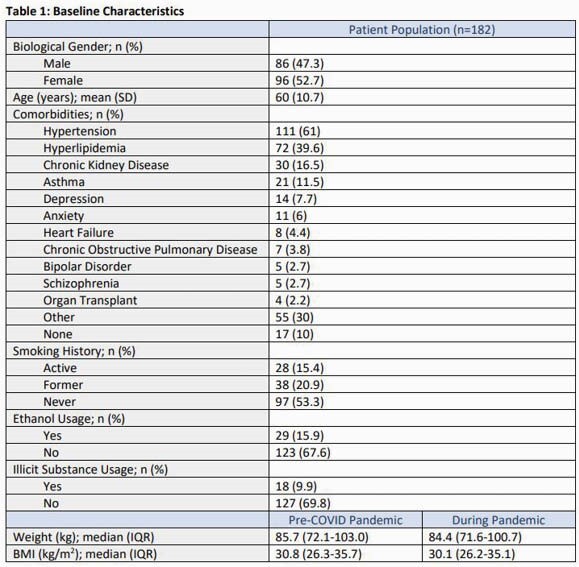

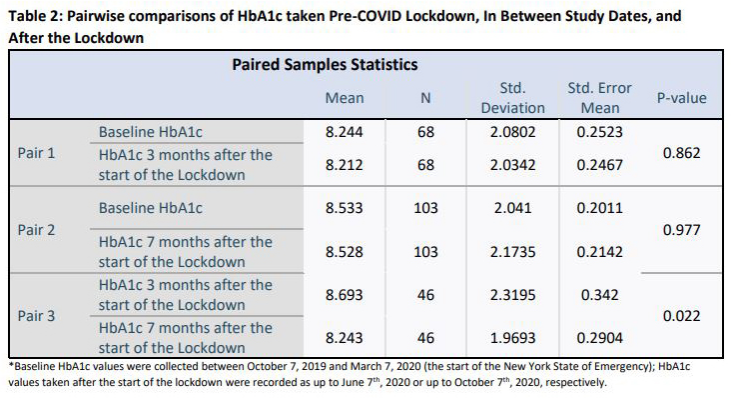

**Conclusion:**

No significance was found in surrogate markers for health maintenance before and after the pandemic. Patients were more likely to keep an appointment after the start of the pandemic and A1c values significantly declined from 3 months to 6 months into the pandemic. Although COVID-19 did not appear to change overall health maintenance of T2DM within our population, our results imply that pandemic measures, such as telehealth appointments, positively affected appointment adherence, which is key to success in this population.

**Disclosures:**

**Jessica E. Yager, MD MPH**, **Abbott Laboratories** (Shareholder)**Amgen Inc** (Shareholder)**Becton Dickenson & Co** (Shareholder)**Edwards Lifesciences Corp** (Shareholder)**Gilead Sciences, Inc.** (Grant/Research Support, Recipient of FOCUS grant)

